# Dr. George Gwynne Bird

**Published:** 1910-05-28

**Authors:** 


### Obituary.

On May 14 died Dr. George Gwynne Bird, of 14 Warwick Avenue, W. He was a student of St. Mary's Hospital, and entered the profession in 1872, when he took his diploma of M.R.C.S. He was anæsthetist to the Female Lock Hospital and a certifying factory surgeon.

